# Supplementary material for: Spectroscopic signature of obstructed surface states in SrIn2P2
Source: Nat Commun. 2023 May 22;14:2905. doi: 10.1038/s41467-023-38589-0 (PMC10203355; doi:10.1038/s41467-023-38589-0)
Supplement: Supplementary file 1 — Supplementary Information [file 41467_2023_38589_MOESM1_ESM.pdf]

Supporting Information

# Spectroscopic signature of obstructed surface states in $SrIn_2P_2$

Xiang-Rui Liu[1,2]*, Hanbin Deng[1,2]*, Yuntian Liu[1,2]*, Zhouyi Yin[1], Congrun Chen[1], Yu-Peng Zhu[1,2], Yichen Yang[3], Zhicheng Jiang[3], Zhengtai Liu[3], Mao Ye[3], Dawei Shen[3], Jia-Xin Yin[4], Kedong Wang[1], Qihang Liu[1,2,5†], Yue Zhao[1,2†], Chang Liu[1,2†]

*[1]Shenzhen Institute for Quantum Science and Engineering (SIQSE) and Department of Physics, Southern University of Science and Technology (SUSTech), Shenzhen, Guangdong 518055, China*
*[2]International Quantum Academy, Shenzhen, Guangdong 518048, China*
*[3]State Key Laboratory of Functional Materials for Informatics, Shanghai Institute of Microsystem and Information Technology, Chinese Academy of Sciences, Shanghai 200050, China*
*[4]Department of Physics, Princeton University, Princeton, New Jersey 08544, USA*
*[5]Guangdong Provincial Key Laboratory of Computational Science and Material Design, Southern University of Science and Technology, Shenzhen 518055, China*

*These authors contribute equally to this work.
†Corresponding authors.
E-mail: liuqh@sustech.edu.cn, zhaoy@sustech.edu.cn, liuc@sustech.edu.cn

# Contents


Section I Sample characterization of $SrIn_2P_2$ single crystals ........ 3

Section II Bond energies between different elements ........ 3

Section III Surface regions with 2*a*-spacing and disordered stripes ........ 4

Section IV STM topography scanned at different biases ........ 5

Section V Evolution of the STS spectra and negative differential conductance (NDC) in the presence of point defects ........ 6

Section VI STM and STS results on regions with different local doping ........ 7

Section VII *dI*/*dV* maps scanned at different biases ........ 8

Section VIII Peak energy distribution and apparent height in STM topography ........ 9

Section IX Mechanism of negative differential conductance and its relation to the spatially-localized surface state ........ 10

Section X ARPES constant energy contours ........ 12

Section XI ARPES $k_z$ dispersion map ........ 14

Section XII ARPES *E*-*k* cuts along the $\bar{K}$-$\bar{\Gamma}$-$\bar{K}$ and $\bar{M}$-$\bar{\Gamma}$-$\bar{M}$ directions ........ 14

Section XIII Brillouin zone and band folding under surface reconstruction; surface energy of different reconstructions ........ 16

Section XIV Surface states on different (0001) terminations of $SrIn_2P_2$ ........ 17

Section XV Calculated partial charge density on different (0001) terminations of $SrIn_2P_2$ ........ 17

Section XVI Explanation of the discrepancy between STM-observed and DFT-calculated height difference between neighboring In atoms ........ 19

References ........ 21

# I Sample characterization of $SrIn_2P_2$ single crystals

We performed systematic characterization on $SrIn_2P_2$ single crystals. The x-ray diffraction result clearly resolves the (00*l*) diffraction peaks (Fig. S1a), while the core-level photoemission spectrum reveal the occupied 4*d* orbitals of the In atoms and the 4*s* orbital of the Sr atoms (Fig. S1b). Both measurements indicate the high quality of our crystals.

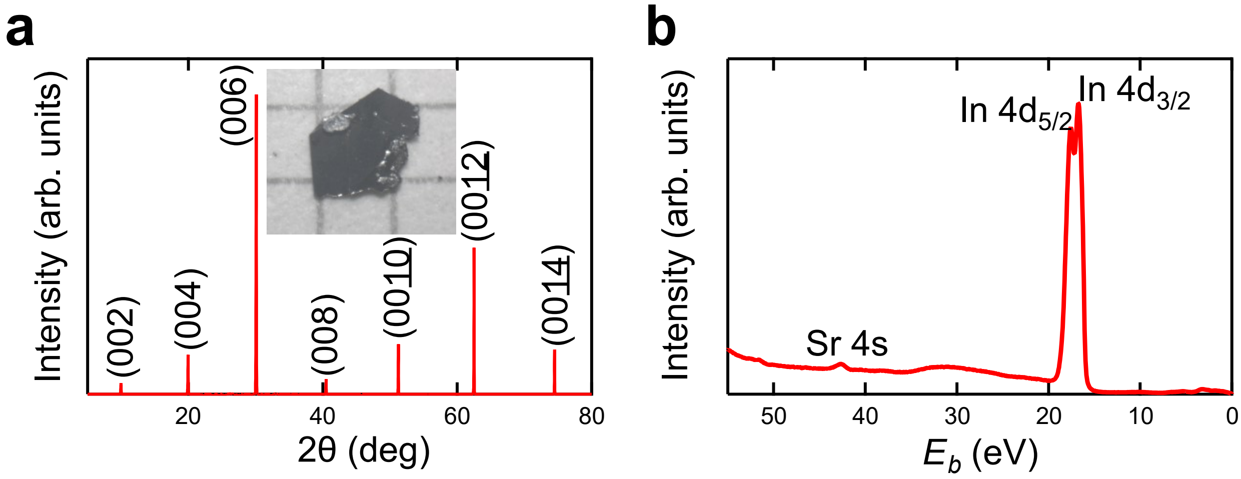


**Fig. S1 Sample characterization of $SrIn_2P_2$ single crystals. a** Single crystal XRD results of the (0001) plane of $SrIn_2P_2$. Inset shows a typical single crystal placed against a millimeter grid. **b** Core level photoemission spectra taken on an *in-situ* cleaved sample.

# II Bond energies between different elements

**Table S1 DFT-calculated exfoliation energy required to form the surface by breaking different bonds.**

| Broken chemical bonds | Exfoliation energy (meV/Å$^2$) |
|---|---|
| In-In | 153 |
| In-P | 309 |
| Sr-P | 213 |

Besides the half-unit-cell height between neighboring terraces found in our STM topographic maps (Fig. 2b in the main text), the cleavage of $SrIn_2P_2$ is confirmed to be happening on the (0001) plane between two In atoms of two adjacent QLs (which cuts through the original OWCCs) based on our calculation of bond energies between different atoms (Table S1). This calculation is done using the method described in Ref. 1. According to our results, the exfoliation energy of the surface

corresponding to the In-In bonds is the smallest, which indicates that the cleavage is most likely to occur between two In atoms.

## III Surface regions with 2*a*-spacing and disordered stripes

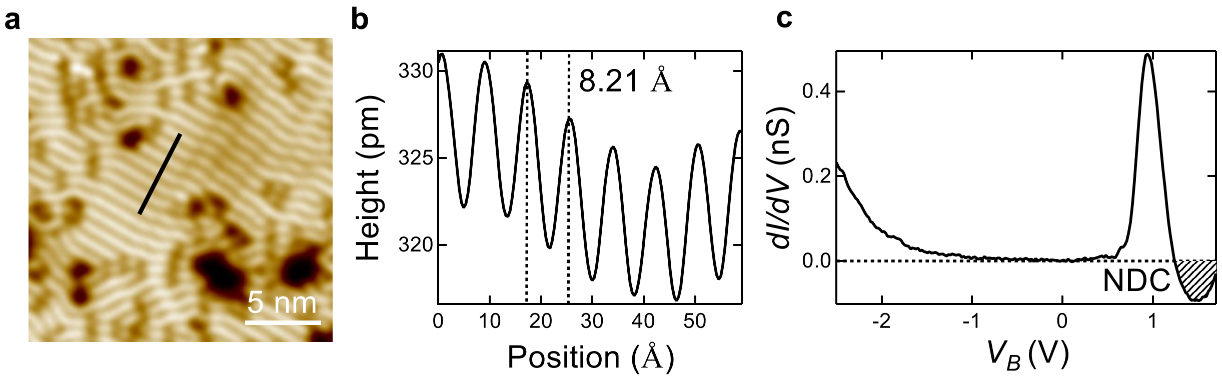


**Fig. S2 Ordered regions with 2*a*-spacing stripes. a** STM topography scanned on a space region different from that in Fig. 2c of the main text, showing 2*a*-spacing stripes. Scanning parameter: $V_B$ = 1 V; $I_t$ = 100 pA. (b) Corrugation curve along the black line shown in **a**. Height modulation with 8.21 Å period indicates stripes with 2*a* spacing ($a$ = 4.09 Å). **c** STS spectra taken on the region in **a**, which also resolves a strong peak centered at $V_B$ = 1.0 V followed by a negative differential conductance (NDC) at higher biases (shaded region).

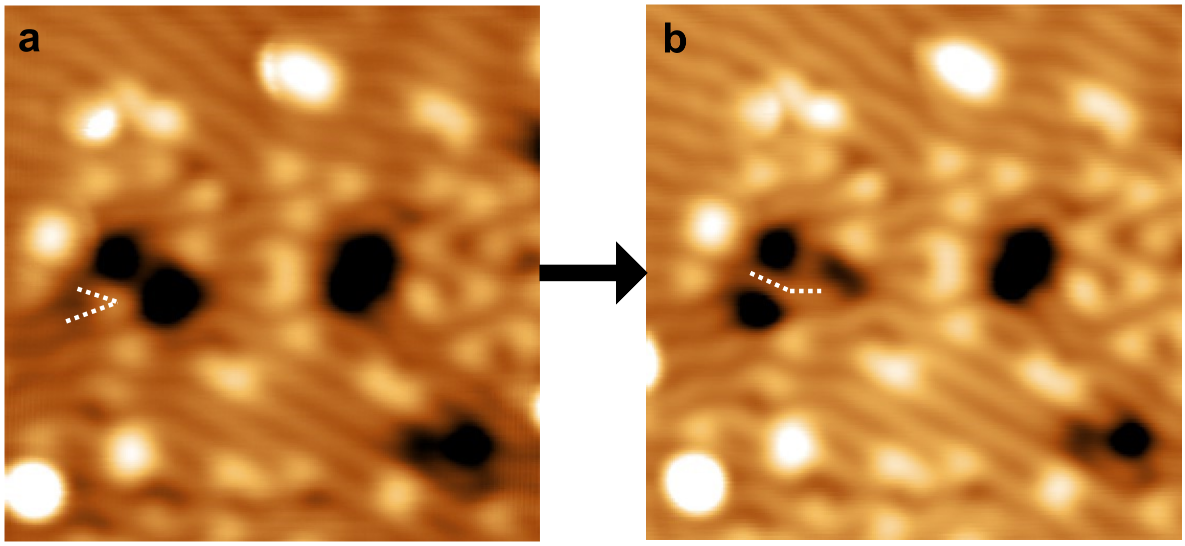


**Fig. S3 Disordered stripes manipulated by STM tip. a**,**b** Two topographic images ($V_B$ = -1 V) obtained on the same field of view before (**a**) and after (**b**) a large-bias-voltage (-2.6 V) STS test. The dotted white lines indicate the directions of the modified stripes.

Apart from the $\sqrt{3}\times 1$ reconstructed regions described in the main text, we also observed stripe-patterned ordered region with $2a$-spacing on the (0001) cleavage plane of $SrIn_2P_2$, as shown in Fig. S2. Similar to the case on regions with $\sqrt{3}a$-spacing stripes, a strong *dI*/*dV* peak followed by negative differential conductance is observed.

In the $\sqrt{3}\times 1$ reconstructed regions, domains of several micrometers with stripes along all three directions can be observed on the sample surface, indicating their energy degeneracy. We can hardly modify the orientation of the stripes within the domain once it is cleaved. However, near the domain boundaries, the disordered stripes may response to a large bias voltage. Fig. S3 shows two topographic images obtained on the same field of view before and after an STS measurement with a large bias voltage, where the direction of a particular stripe (marked by the dotted white lines) is modified because of the application of a large bias.

## IV STM topography scanned at different biases

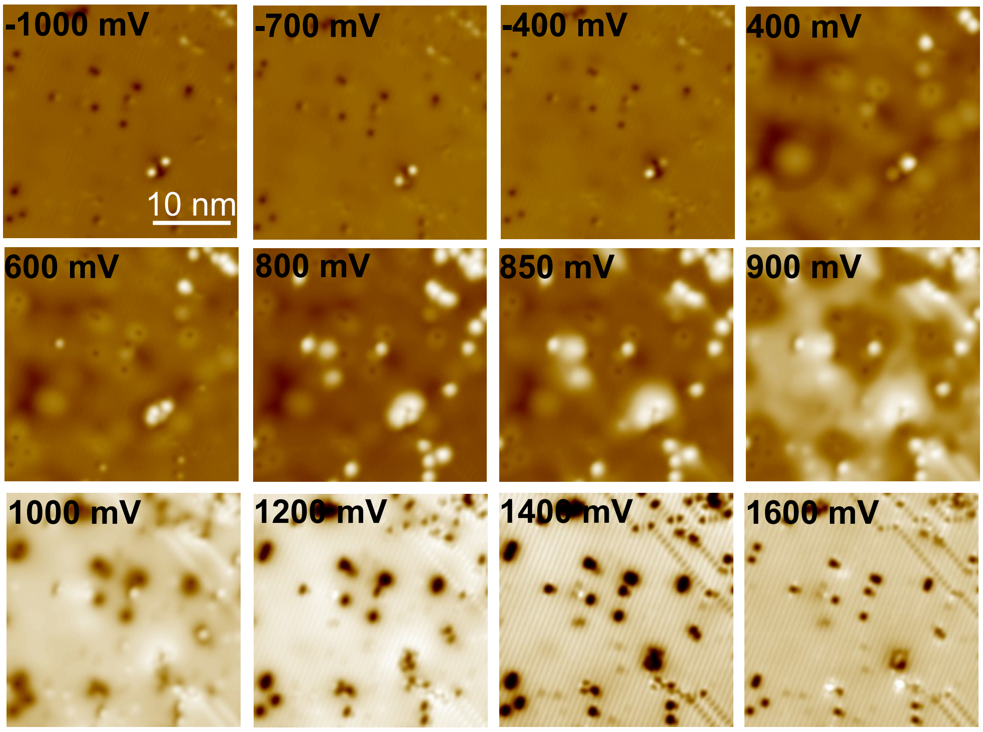


**Fig. S4 STM topography scanned at different bias voltages on the same region as shown in Fig. 2 in the main text.** The scan current is set to be $I_t$ = 500 pA. The stripe-like topography shows no obvious differences when $V_B$ increases from -1.0 to 0.1 V, but changes dramatically from 0.8 to 1.2 V, as it is seemingly covered by a new layer of electron cloud.

We performed systematic STM topography study on the (0001) cleavage plane of $SrIn_2P_2$. The topography shows no obvious change from $V_B$ = -1 to 0.1 V, but shows a dramatic change from $V_B$ = 0.8 to 1.2 V, as shown in Fig. S4.

## V Evolution of the STS spectra and negative differential conductance (NDC) in the presence of point defects

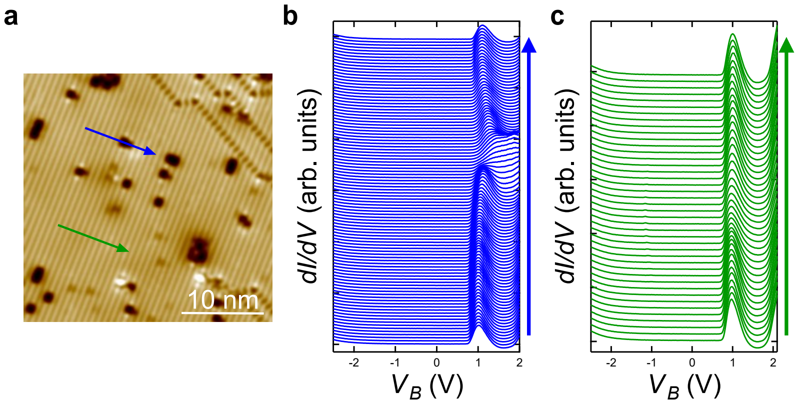


**Fig. S5 Evolution of the STS spectra in the presence of point defects. a** STM topography scanned with the same parameters on the same region as shown in Fig. 2c of the main text. **b** STS line cuts along the blue arrow in **a**. Both the strong peak centered at $V_B$ = 1.0 V and the NDC that follows at higher biases disappear near a point defect of the surface (black region in **a**). **c** STS line cuts along the green arrow in **a**, cutting through pristine regions on the surface.

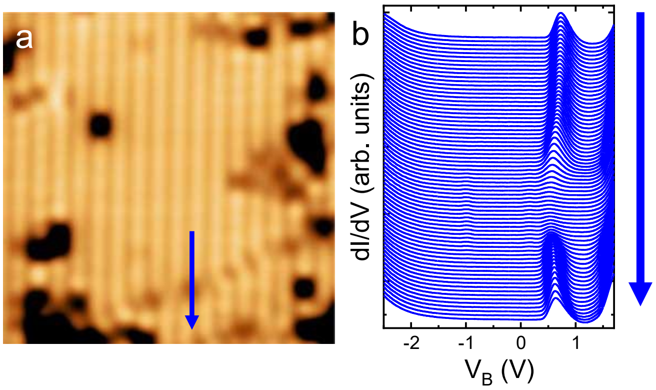


**Fig. S6 Another set of data showing how a point defect destroys the STS peak and the following negative differential conductance. a** STM topography scanned on an ordered $\sqrt{3} \times 1$ reconstructed region other than that shown in Fig. 2 of the main text ($V_B$ = 1.3 V, $I_t$ = 500 pA). **b** STS line cuts along the blue arrow in **a**. Both the strong peak centered at $V_B$ = 0.7 V and the NDC that follows at higher biases disappear near a point defect of the surface.

We performed systematic STS study on the (0001) cleavage plane of $SrIn_2P_2$. The strong *dI*/*dV* peak and following negative differential conductance on the $\sqrt{3}\times 1$ reconstructed region is found to be volatile against surface defects and contaminations. As shown in Figs. S5 and S6, a point defect is enough to destroy the STS peak and the negative differential conductance.

## VI STM and STS results on regions with different local doping

We found via repeated STS measurements that the energy position of the strong *dI*/*dV* peak depends on the local doping level close to the STM tip. While 1.0 eV might be a little far away from the DFT prediction, there are regions on the sample where the peak locates at 0.7 eV above $E_F$, which coincides with the energy location of the DFT-predicted upper branch of the surface state. Fig. S7 shows STS spectra on another surface region with a lower local doping level. In the ordered $\sqrt{3}\times 1$ reconstructed region, the results are consistent with those in Fig. 2, except for a 0.3 eV upshift of the Fermi level (0.3 eV downshift of the peak position). The strong peak is now centered at ~ 0.7 eV, which is closer to the energy position of the DFT-calculated unoccupied surface state. Thus, the discrepancy between the STS spectra shown in Fig. 2 and the DFT calculation (which agrees well with ARPES results) likely comes from a Fermi level shift that depends on the local doping level. Such pronounced shift of doping level also suggests that such strong *dI*/*dV* peak is of surface nature.

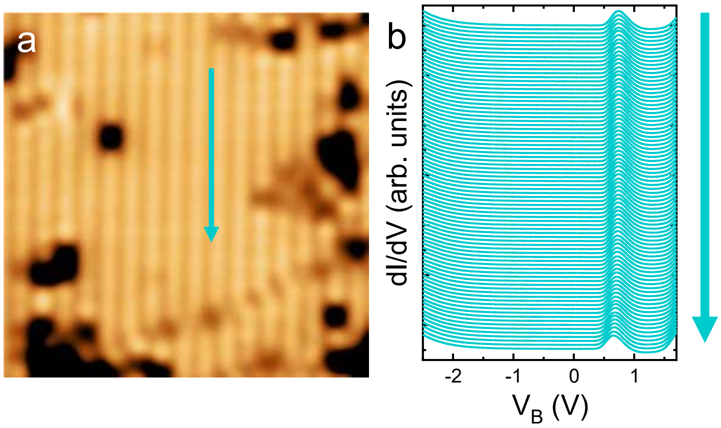


**Fig. S7 Ordered $\sqrt{3}\times 1$ reconstructed region with a lower local doping level. a** STM topography scanned on ordered $\sqrt{3}\times 1$ region with a lower local doping level ($V_B$ = 1.3 V, $I_t$ = 500 pA). **b** STS line cuts along the cyan arrow in **a**. The strong STS peak is centered at $V_B$ = 0.7 V instead of $V_B$ = 1.0 V in the case of Fig. 2 of the main text. The NDC following the peak is also clearly visible.

## VII *dI/dV* maps scanned at different biases

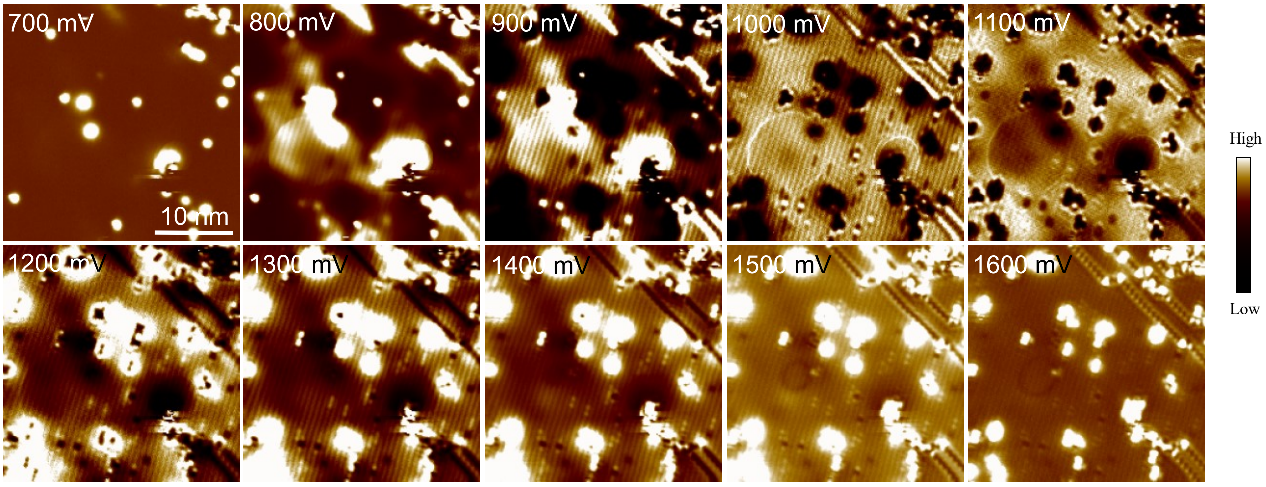


**Fig. S8 *dI/dV* maps scanned at high biases (700 – 1600 mV) on the same region as shown in Fig. 2c of the main text.** The color scale is set such that the difference between $(dI/dV)_{\rm max}$ (white) and $(dI/dV)_{\rm min}$ (black) is constant for all panels. In other words, the same color contrast in each panel represents the same difference in the absolute values of the differential conductance. The junction is set to be $V_b$ = 1.6 V, $I_t$ = 500 pA.

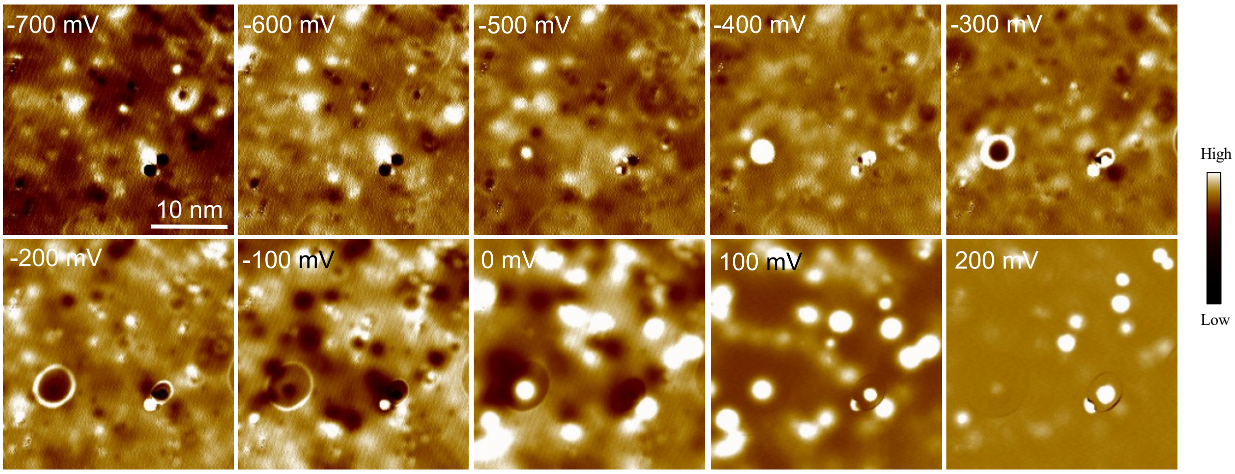


**Fig. S9 *dI/dV* maps scanned at low biases (-700 – 200 mV) on the same region as shown in Fig. 2c of the main text.** The color scale is set in the same manner as Fig. S5. The junction is set to be $V_b$ = -700 mV, $I_t$ = 500 pA.

We performed systematic *dI*/*dV* maps on the (0001) cleavage plane of $SrIn_2P_2$ over a wide range of biases, as shown in Figs. S8 and S9. For biases close to the energy of STS peak, the corresponding *dI*/*dV* maps show ordered stripes-like feature.

## VIII Peak energy distribution and apparent height in STM topography

From our data, all the defect-free area on $SrIn_2P_2$ shows similar NDC following strong STS peak behavior, indicating a particularly unusual origin. It is worth noting that near the STS peak energy, dramatic height steps (~ 300 pm) appear abruptly at the originally flat region between areas with slightly different doping. As shown in Fig. S10, the field of view shows a relatively flat surface at the defect-free area (Fig. S10c) at bias voltage $V_B$ = 1.6 V. The STS peak positions at the defect-free area are plotted in Fig. S10a. When we obtain the topographic image at $V_B$ = 0.9 V on the same area (Fig. S10b), those regions with the same STS peak energy (0.9 V, magenta regions in Fig. S10a) experience a sudden uplift of ~ 300 pm, as if there appears a floating cloud.

The STM topographic image is a combination of surface corrugation and surface electronic state[2]. Such an abrupt height change near the STS peak energy marks a sudden appearance of an extremely high level of surface charge density. Although it is difficult to quantitatively evaluate experimentally how much larger the LDOS peak is compared to other conventional surfaces, we are confident that the manifestation of NDC provides valuable evidence for the presence of a ubiquitous structure for resonant tunneling beneath the STM tip – a surface state that is likely localized both in energy and in space along the *z* direction. This is consistent with our DFT calculation, that the surface state originates from the OWCCs where no atom resides. The electron cloud of these obstructed charge densities is much closer to vacuum than real atoms, making it the most possible omnipresent structure for resonant tunneling in our study.

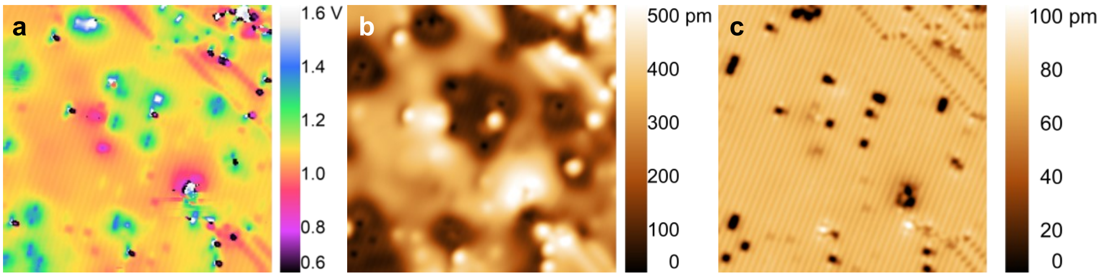


**Fig. S10 Energy distribution of the STS peak induces large difference of the apparent height in the topographic image. a** A map showing the energy position of the STS peaks of the defect-free area, indicating the doping difference of the sample surface. Data is extracted from Fig. S8 by fitting the *dI/dV* vs. *V* plot with Gaussian equations in each pixel. **b**,**c** Topographic images of the same field of view at (**b**) $V_B$ = 0.9 V, $I_t$ = 500 pA, and (**c**) $V_B$ = 1.6 V, $I_t$ = 500 pA, showing an abrupt rise in elevation ($\Delta z \approx$ 300 pm) of areas with slight doping difference.

## IX Mechanism of negative differential conductance and its relation to the spatially-localized surface state

In this section, we explain in detail why a negative *dI/dV* can appear on the STS spectrum.

Negative differential conductance (NDC) is an uncommon property where an increase in bias voltage results in a decrease in current. It occurs when a resonant tunneling structure is present, where electrons can tunnel through potential barriers at certain energy levels with complete transmission. NDC is a key characteristic of resonant tunneling.

In transport measurements, such a phenomenon is usually observed in resonant tunneling diodes made up of various heterostructures, since the presence of spatially separated energy levels is a prerequisite. Several types of heterostructures have been observed to host NDC because of resonant tunneling, ranging from quantum well structures of semiconductors (AlAs/InGaAs, Si/SiGe, etc.) to Dirac fermions with a thin dielectric spacer (graphene/h-BN/graphene)[3].

In STM measurements, NDC can provide valuable insights into the underlying resonant tunneling structure between the tip and the sample surface. Observation of NDC following a strong STS peak in a single crystal surface are particularly rare, indicating both a high local density of states (LDOS) and a resonant tunneling structure in the tunneling geometry near the high LDOS energy level. To our best knowledge, such observations have only been reported in the following cases:

(1) Adatoms/molecules that are sparsely distributed on the sample surface, forming a metal-adatom/molecule-metal junction. In this case, resonant tunneling between the tip and the adatom/molecule orbitals gives rise to the STS peak and the associated NDC[4,5]. When more than one molecule is involved in the tunneling process (a chain of molecules), NDC could be also a result of the resonant tunneling from multiple molecular orbitals.

(2) Two-dimensional materials where surface states with both narrow energy localization and spatial localization could be available. Examples of such resonant tunneling include

a) layer-polarized van Hove singularity in bilayer graphene[6];

b) magnetic field-induced edge states at the npn junction boundaries in graphene[7];

c) the sublattice and layer localization of the nearly flat bands in ABC trilayer graphene[8].

For all the cases above, the NDC-following-STS-peak behavior is not global. It occurs only at atomic sites where a resonant tunneling structure exists: adatom/molecule sites with orbital states in Case 1; the BT atomic site where the layer polarized van Hove singularity can be probed in Case 2a;

the boundary of the npn junction where the Landau levels reside in Case 2b; and the A1 atomic site with the sublattice and layer localized nearly flat band in ABC trilayer in Case 2c.

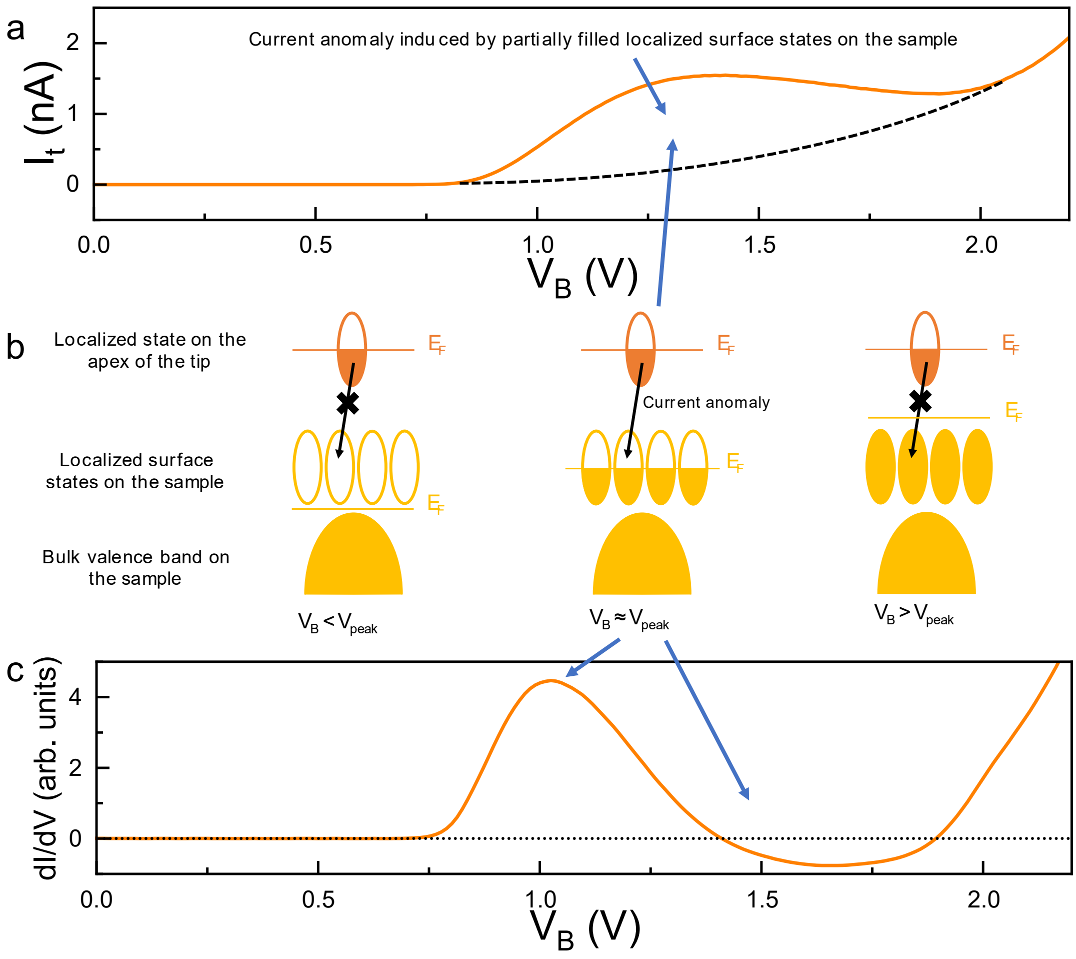


**Fig. S11 Due to the localized states on the apex of the STM tip, filling of the localized surface states on the sample induces tunneling current anomaly and negative differential conductance (NDC). a** Measured *I* vs. *V* curve on the surface of $SrIn_2P_2$. The dotted black line is the presumed tunneling current without the localized-state resonance peak. **b** Illustration of tunneling current anomaly induced by local-state resonance. From left to right: When $V_B < V_{peak}$ ($V_B$ being the bias voltage), the surface state peaks are not filled, no tunneling current anomaly is present. When $V_B \approx V_{peak}$, the surface state peaks are partially filled, tunneling current anomaly emerges. When $V_B > V_{peak}$, The surface state peaks are fully filled, there is again no tunneling current anomaly. The tunnelling current decreases with increasing bias voltage, giving rise to NDC. **c** Measured *dI/dV* versus *V* plot on the surface of $SrIn_2P_2$. Due to the current anomaly induced by local-state resonance, the phenomenon of a peak following by a NDC appears.

Fig. S11 describes the detailed process of resonant tunneling through the electron cloud originating from the OWCCs. The apex of the STM tip is a protruding atom (an atom that projects outward). Its spatial constraint will introduce localized electronic states like those in a quantum well. In ordinary conditions, no localized surface states reside outside the sample surface, and the tunneling current from the localized states on the tip contributes negligibly to the tunneling current compared with that from the tip's metallic states (states that come from the element of the tip itself, like W, Pt or Ir). Therefore, Bardeen's formula is valid:

$$\frac{dI}{dV} \propto \int_{-\infty}^{\infty} f'(E_F + \varepsilon)\rho_s(E_F - eV + \varepsilon)\, d\varepsilon \approx \rho_s(eV).$$

However, on $SrIn_2P_2$, spatially-localized surface states situate higher than the sample's itinerant states in real space, deep into the vacuum region where no atoms reside, described as the obstructed Wannier charge centers. The overlap between the protruding localized states on the sample and the protruding localized states on the tip strongly enhances the tunneling matrix element. Therefore, the part of the tunneling current from the tip's localized states can no longer be ignored. Instead, it dominates the measured tunneling current. The tunneling current can now be approximated as

$$I \propto \int_{0}^{eV} \rho_s(E_F - eV + \varepsilon)\rho_T(E_F + \varepsilon)\, d\varepsilon.$$

Here the tip's density of states $\rho_T(E)$ is assumed to have a localized state near $E_F$ [2,4].

Without the tunneling channel from the apex of the tip, the *I-V* curve should be monotonic, as shown by the dotted black line in Fig. S11a. When the two localized states line up for the resonant tunneling at $V_B \approx V_{peak}$, the measured *I-V* curve deviates from a monotonic one because of the dramatically enhanced tunneling. A current anomaly takes place. With such an anomaly in the *I-V* curve, a strong peak followed by an NDC appears in the *dI/dV-V* plot.

In summary, the NDC following a strong peak observed all over the defect-free area provides insightful hint for the existence of an "obstructed" electronic structure in $SrIn_2P_2$.

## X ARPES constant energy contours

ARPES constant energy contours taken at 76 and 90 eV photon energies are shown in Figs. S12 and S13. The $k_z$s of different photon energies are calculated via $k_z = \sqrt{2m/\hbar^2(E_k \cos^2\theta + V_0)}$ with inner potential $V_0$ = 14 eV (See section XI for more details). The map collected at $h\nu$ = 76 eV corresponds to $k_z$ = 4.741 Å$^{-1}$ (~0.9 $\pi/c$) at $\bar{\Gamma}$, while the map collected at $h\nu$ = 90 eV corresponds to $k_z$

= 5.114 Å$^{-1}$ (~π/$c$) at $\bar{\Gamma}$ ($c$ = 17.812 Å). The constant energy contours within 1.0 eV binding energies show isotropic circular pattern, while those with binding energies from 1.5 to 2.5 eV show six-fold patterns. The constant energy contours in the two maps show quite different patterns though their $k_z$ value is close to each other. We ascribe such behavior to the matrix element effect of the bulk states.

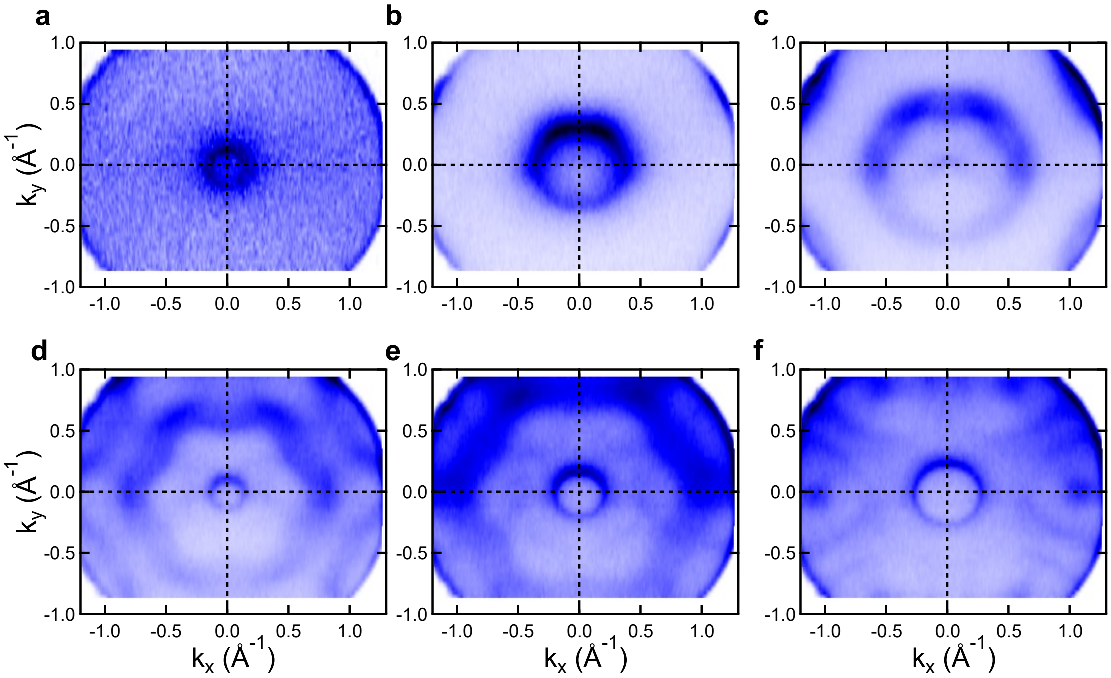


**Fig. S12 ARPES constant energy contours taken at $h\nu$ = 76 eV.** $k_x$ is aligned along $\bar{K}$-$\bar{\Gamma}$-$\bar{K}$, and $k_y$ is aligned along $\bar{M}$-$\bar{\Gamma}$-$\bar{M}$. The binding energies of **a** to **f** are 0, 0.5, 1.0, 1.5, 2.0 and 2.5 eV, respectively.

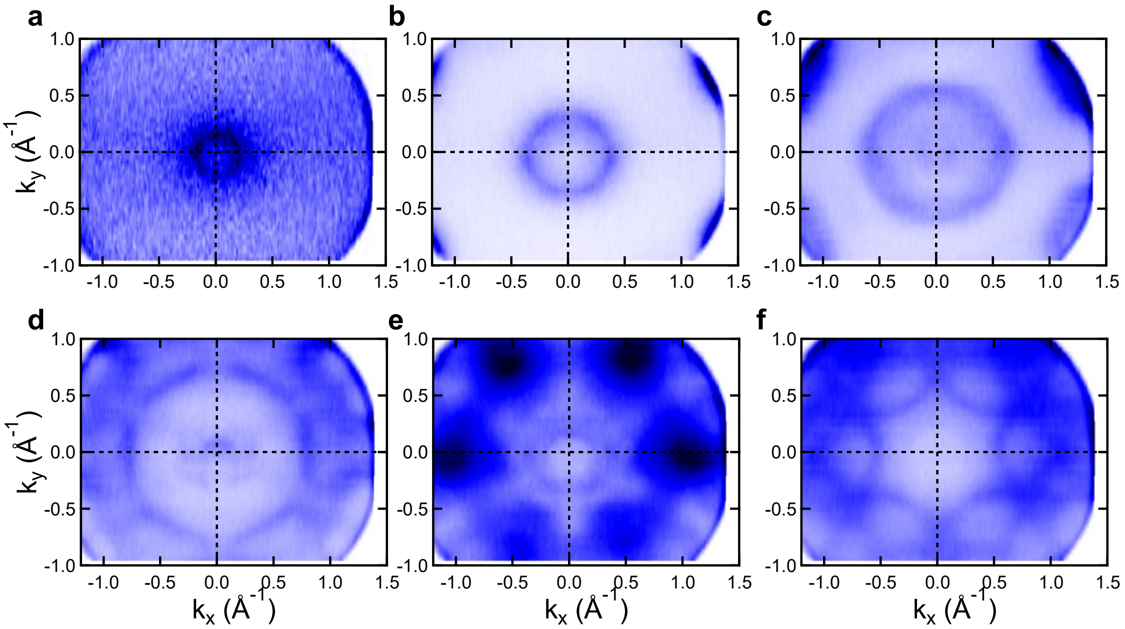


**Fig. S13 ARPES constant energy contours taken at $h\nu$ = 90 eV.** $k_x$ is aligned along $\bar{K}$-$\bar{\Gamma}$-$\bar{K}$, and $k_y$ is aligned along $\bar{M}$-$\bar{\Gamma}$-$\bar{M}$. The binding energies of **a** to **f** are 0, 0.5, 1.0, 1.5, 2.0 and 2.5 eV, respectively.

## XI ARPES $k_z$ dispersion map

A systematic photon-energy-dependent map from $h\nu$ = 50 to 100 eV were performed. The in-plane momentum is aligned along the $\bar{\Gamma}$ - $\bar{K}$ direction, and $k_z$ is calculated via $k_z = \sqrt{2m/\hbar^2(E_k\cos^2\theta + V_0)}$ with inner potential $V_0$ = 14 eV. Figs. S14a-c show constant energy contours at $E_b$ = 0.2, 1.0 and 1.4 eV, respectively. The two vertical lines correspond to the surface states seen in Fig. 3 of the main text, showing no $k_z$ dispersion within $0 < E_b < 1.4$ eV, proving unambiguously their two-dimensional nature. On the other hand, the BS2 band shows $k_z$ dispersion with a periodicity of $2\pi/c'$ ($c' = c/2$) at $E_b$ = 1.0 eV (Fig. S14b), which further confirms its bulk nature. This quasi $k_z$ periodicity of the BS2 band doubles the size of the Brillouin zone projection along the *c* axis, which originates from the fact that two quintuple layers (QLs) stack in one unit cell (Fig. 1d of the main text). To reveal the $k_z$ dispersion of the bulk bands at $\bar{\Gamma}$ more clearly, a $E$-$k_z$ cut taken from the $k_z$ map at $k_{\Gamma\text{-K}} = 0$ was shown in Fig. S14d. The red dashed line represents the DFT calculated result of BS1 along Γ-A after an energy shifting of 0.25 eV upward, which agrees well with the observed dispersion in the cut.

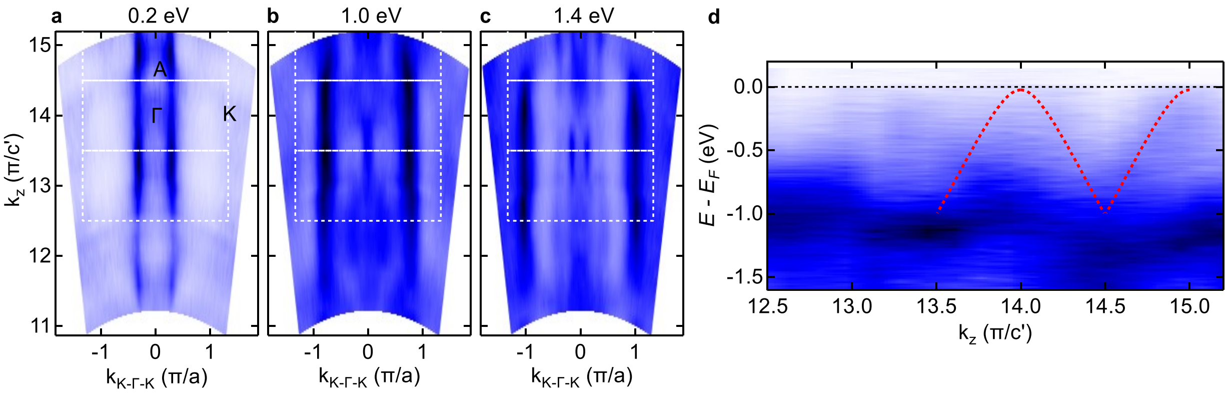


**Fig. S14 ARPES $k_z$ dispersion maps. a-c** Constant energy contours at $E_b$ = 0.2, 1.0 and 1.4 eV in a $k_z$-$k_{\Gamma\text{-K}}$ map with a photon energy range of $50 < h\nu < 100$ eV. Here $c' = c/2$. White dashed lines indicate the projected Brillouin zone. **d** $E$-$k_z$ cut at $k_{\Gamma\text{-K}} = 0$. The red dashed line represents the calculated out-of-plane dispersion of the topmost valence band along the Γ-A direction after an energy upshift of 0.25 eV.

## XII ARPES *E*-*k* cuts along the $\bar{K}$-$\bar{\Gamma}$-$\bar{K}$ and $\bar{M}$-$\bar{\Gamma}$-$\bar{M}$ directions

To further prove that the linear band observed by ARPES (Fig. 3 in the main text) is indeed the lower branch of the surface state, we show in Figs. S15 and S16 that traces of this surface state extend

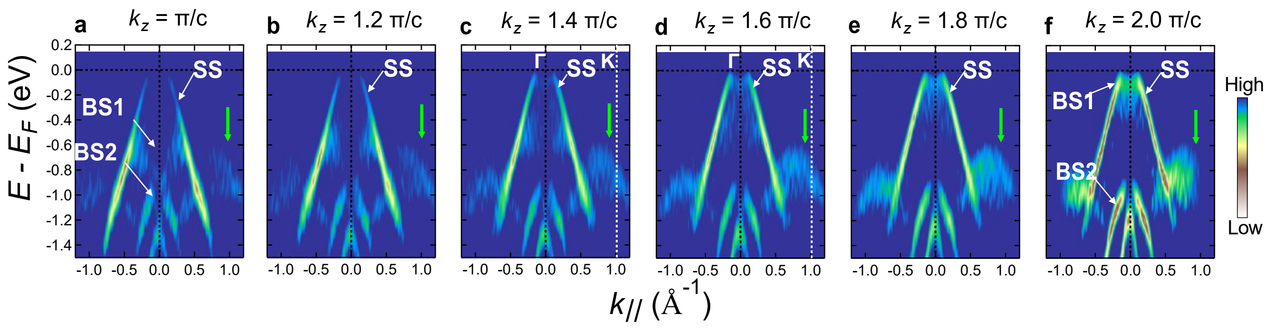


**Fig. S15 Second order curvature analysis of band structure of $SrIn_2P_2$ at different $k_z$s. a-f** Second order curvature analysis along energy distribution curves for the spectra in Fig. 3a-f of the main text. Here we remove the red lines that represent the DFT-calculated bands to make BS1 and BS2 clearer. White arrows: dispersion of the OSS-derived surface state (SS) and the bulk bands BS1 and BS2; green arrows: tail of the SS outside the hole-pocket region. Note that the $\bar{K}$ point is marked in **c** and **d**, indicating that the residue intensity of the surface state extends to $\bar{K}$.

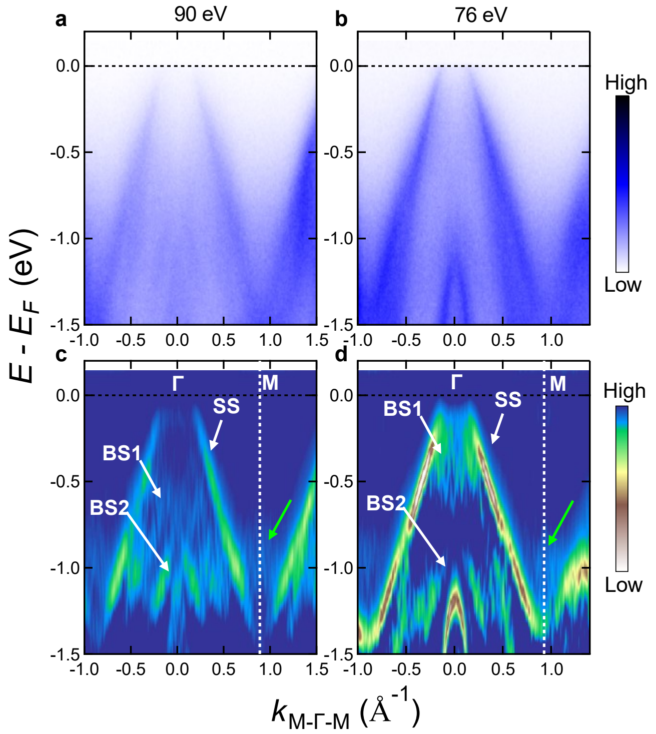


**Figure S16 ARPES *E*-*k* cuts at different photon energies along $\bar{M}$-$\bar{\Gamma}$-$\bar{M}$. a,b** ARPES *E*-*k* cuts along the $\bar{M}$-$\bar{\Gamma}$-$\bar{M}$ direction at $h\nu$ = 90 and 76 eV, which correspond to $k_z$ = 4.741 Å$^{-1}$ (~0.9 $\pi/c$) and 5.114 Å$^{-1}$ (~$\pi/c$), here $c$ = 17.812 Å. **c,d** Second order curvature analysis along the energy distribution curves of the spectra in **a**,**b**. White arrows: dispersion of the OSS-derived surface state (SS) and the bulk bands BS1 and BS2; green arrows: tail of the SS outside the hole-pocket region. Note that the $\bar{M}$ point is marked in **c** and **d**, indicating that the residue intensity of the surface state extends to $\bar{M}$.

all the way to the $\bar{K}$ and $\bar{M}$ points, whose dispersion agrees qualitatively with the DFT results. By applying the second-order curvature analysis to the raw data, extra intensity matching the dispersion of the surface state is clearly visible near the $\bar{K}$ and $\bar{M}$ points, indicated by the green arrows in Fig. S15a-f and Fig. S16c-d. Such dispersion is consistent with our calculation results for the lower branch of the split surface state shown in Fig. 4c of the main text.

## XIII Brillouin zone and band folding under surface reconstruction; surface energy of different reconstructions

To compare the stripes with $\sqrt{3}a$ and $2a$ spacing measured by STM, we built $\sqrt{3}a \times 1a$ and $\sqrt{3}a \times 2a$ supercells to calculate the surface reconstruction. Resulting Brillouin zone folding of the $\sqrt{3}a \times 1a$ supercell is shown in Fig. S17a. One of the $M$ points of the unit cell Brillouin zone is folded to the $\Gamma$ point, while another one is folded to the $M$ point of the supercell Brillouin zone. At the same time, the surface states also increased from two bands per surface of the unit cell to four bands per surface (Fig. S17b-c). After that, the atoms in the top five atomic layers were fully relaxed until the maximal forces on them were less than 0.001 eV/Å. The result of the $\sqrt{3}a \times 1a$ supercell is shown in Fig. 4c of the main text, in which the two In atoms on the topmost surface layer are shifted by 113 pm in the *c* direction, while the total energy is decreased by 26 meV/Å$^2$. For a calculation

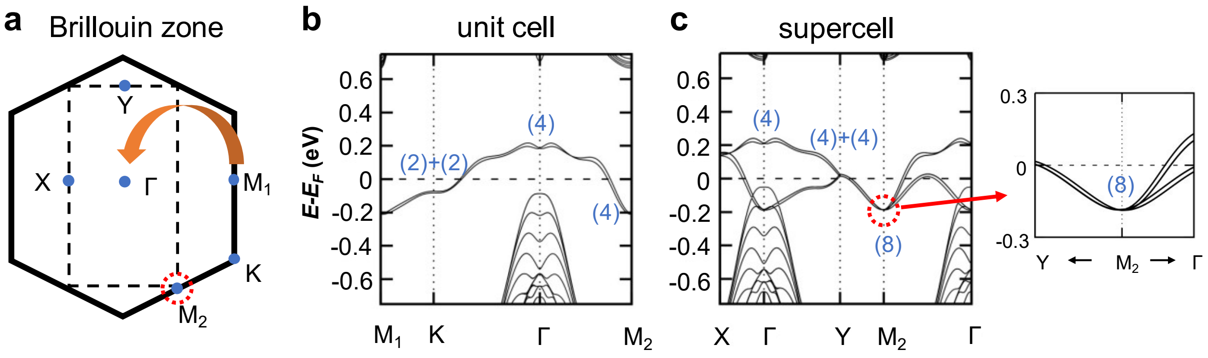


**Fig. S17 Brillouin zone and band folding under surface reconstruction. a** Schematic illustration of the Brillouin zone folding. The solid hexagon is the Brillouin zone of the slab unit cell and the dashed rectangular is the Brillouin zone of the slab $\sqrt{3}a \times 1a$ supercell. **b**,**c** Band structures of the slab unit cell and the slab $\sqrt{3}a \times 1a$ supercell, respectively. The red dashed circles in **a** and **c** and the associated enlarged view (**c**, right) depicts the eight-fold degenerate bands at the $M_2$ point on the boundary of the Brillouin zone after folding.

using the $\sqrt{3}a \times 2a$ supercell, the surface is found to be relaxed to the $\sqrt{3}a \times 1a$ supercell, which shows that the stripes with $\sqrt{3}a$ spacing have the lowest energy and is consistent with the experimental results that most of the surface regions shows $\sqrt{3} \times 1$ reconstruction.

For the non-magnetic material $SrIn_2P_2$ with a termination, time-reversal symmetry will be maintained on the boundary, protecting Kramer's degeneracy at time-reversal invariant momenta (TRIM) of all bands, including the surface state bands. Therefore, the surface bands are 4-fold degenerate at the TRIMs in the slab calculation of Fig. 1e, including 2-fold from the time-reversal-protected Kramer's degeneracy of a single surface, and the other 2-fold from the degeneracy of the top and bottom surfaces. The folded boundary of the Brillouin zone will naturally provide another 2-fold degeneracy of a band, comprising a total of 8-fold degeneracy of the surface bands at the TRIMs on the boundary, such as the $M_2$ point marked in Fig. S17c.

## XIV Surface states on different (0001) terminations of $SrIn_2P_2$

We performed DFT calculations on the surface states of other possible (0001) terminations of $SrIn_2P_2$ which do not cut through the obstructed Wannier charge centers (OWCCs), including the terminations that break the In-P bonds and the Sr-P bonds (Fig. S18b,d). The surface states on all of these terminations show dispersion distinct from the one that breaks the In-In bonds (the one that cuts through the OWCCs), but none of them passes the Fermi level. In these cases, the whole system (bulk + surface) is insulating. These surface states are ordinary surface states, not obstructed surface states.

## XV Calculated partial charge density on different (0001) terminations of $SrIn_2P_2$

As for the surface state observed on the (0001) surfaces of $SrIn_2P_2$ that breaks chemical bonds other than the In-In bond, no OWCC is presented on those surfaces, so at least the charge density is expected to be centered at the surface atoms rather than the OWCCs between atoms. In Fig. S19 we present additional DFT calculations of the partial charge densities on an exemplified cleavage surface without OWCCs (the one that breaks the In-P bonds) and compare it with that on the surface with OWCCs. Compared with the ordinary termination without OWCCs (Fig. S19b), the surface charge of the termination crossing the OWCCs is significantly deviated from the surface atoms (Fig. S19a). Importantly, this deviation is found to be retained after surface reconstruction (Fig. 4 in the main text).

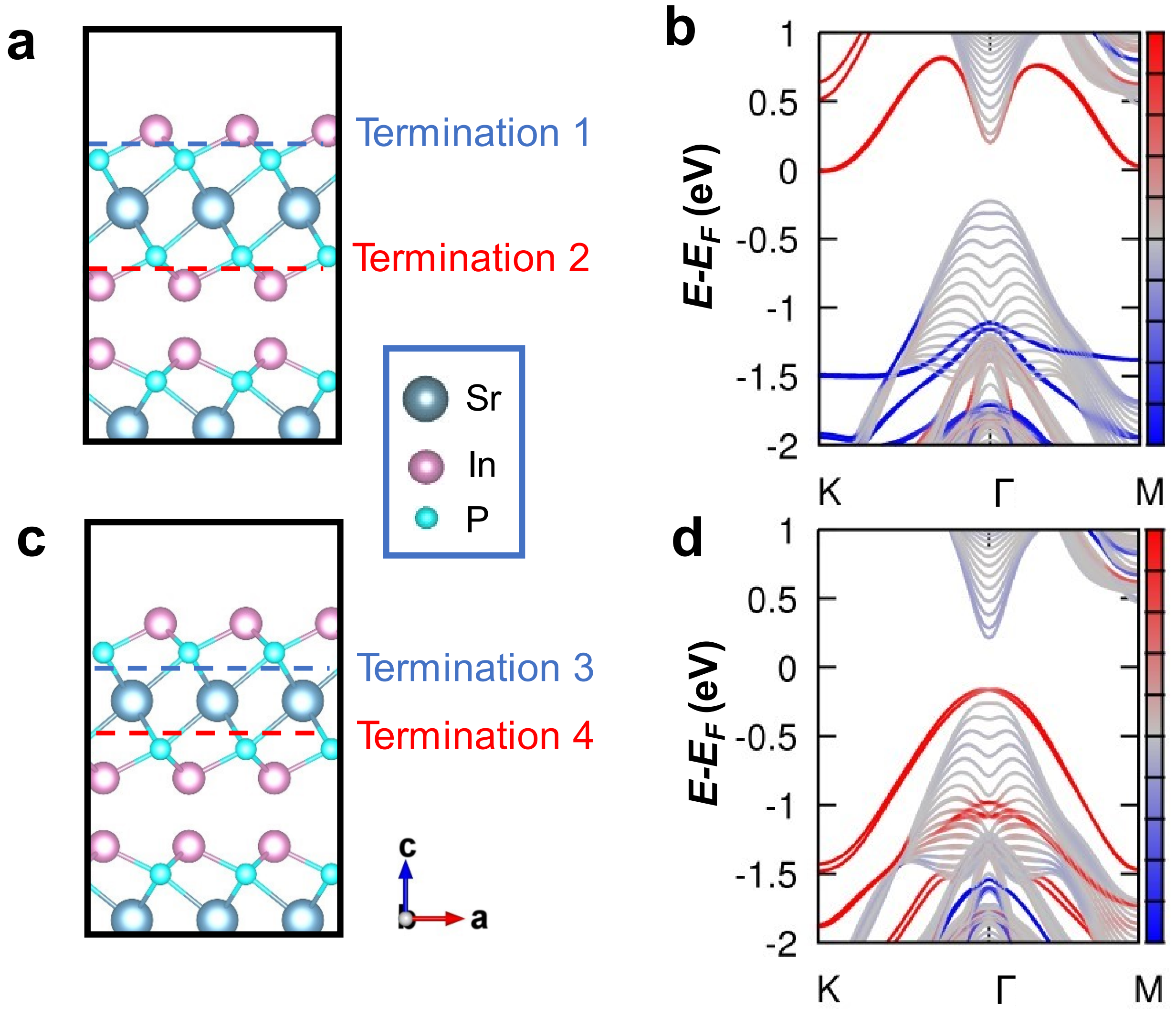


**Fig. S18 Surface states formed on different (0001) terminations of $SrIn_2P_2$. a,c** Schematic illustration of $SrIn_2P_2$ (0001) termination that breaks the (**a**) In-P and (**c**) Sr-P bonds. Terminations 1, 2 and 3, 4 would form on the (0001) cleavage surface, respectively. There are two terminations in each case since the top and bottom interfaces correspond to different terminations. **b**,**d** DFT-calculated (0001) surface states on a $SrIn_2P_2$ slab model with terminations that break (**b**) the In-P bonds and (**d**) the Sr-P bonds, respectively. The blue bands in **b** and **d** represent the projected surface states formed by Terminations 1 and 3, respectively, while the red bands in **b** and **d** represent the projected surface states formed by Terminations 2 and 4, respectively. The gray bands represent the bulk bands.

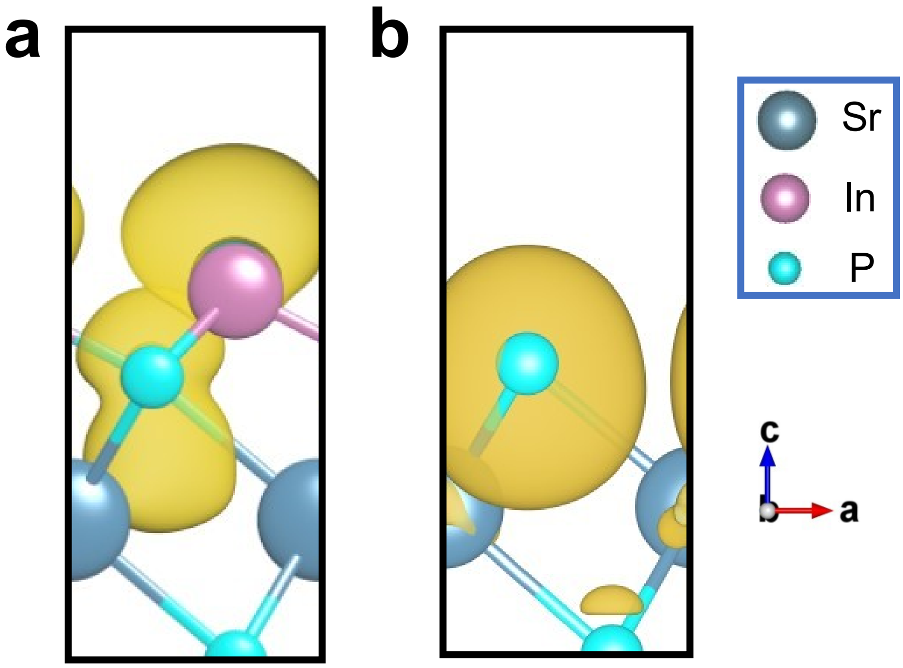


**Fig. S19 DFT calculated partial charge density for different terminations. a** The termination that breaks the In-In bonds, cutting through the OWCCs. **b** The termination that breaks the In-P bonds, without cutting through the OWCCs. The golden regions represent the partial charge density distributions evaluated in the energy range of -0.5 – 0.5 eV comparing to respective Fermi levels.

## XVI Explanation of the discrepancy between STM-observed and DFT-calculated height difference between neighboring In atoms

In Fig. S20 we show further explanation from DFT calculations on why the observed height difference between neighboring In atoms in the $\sqrt{3}a \times 1a$ supercell can be much smaller than the DFT calculated value. The measured height difference in the STM images is close to the height difference between the charge density on the left In atom (golden bubble) and the atomic position of the right In atom (marked as "STM" in Fig. S20), rather than the actual height difference between two surface In atoms (marked as "DFT" in Fig. S20).

The height difference measured in STM is an experimentally-adjustable quantity. The exact value of the height difference depends on the choice of the tunnelling current, as different tunnelling current probes different charge densities. On the other hand, the height difference calculated by DFT is also adjustable. As shown in Fig. S20, the apparent height difference between the DFT charge

density above the lower In atom (golden bubble) and the position of the elevated In atom (pink atom on the right) will change if we choose different charge density values for the bubble. The higher the density, the smaller the bubble, and vice versa. Therefore, a particular tunnelling current we use in the STM measurement effectively probes a particular volume of the bubble, leading to a particular height difference.

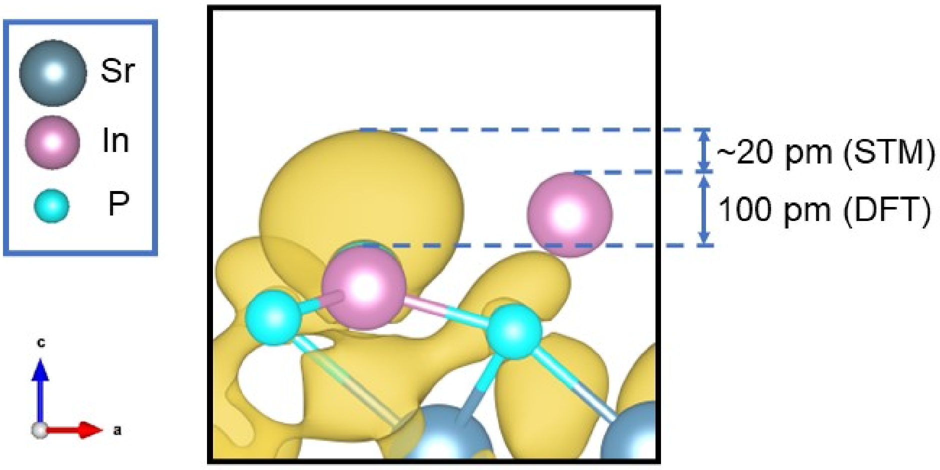


**Fig. S20 Hint from DFT calculation on why the STM-observed height difference between neighboring In atoms is much smaller than the DFT-calculated value.** The figure shows an exemplified charge density contour (golden bubble) surrounding the lower In atom, as well as the actual In atom (pink ball) on the right. What an STM topographic image observed is roughly the height difference between the left contour and the right atom (marked as "STM"), while the DFT-determined height difference between atoms is the actual height difference between the two pink balls, marked as "DFT". These two quantities are unrelated, and need not be comparable in values.

## References


1 Jung, J. H., Park, C.-H., & Ihm, J. A rigorous method of calculating exfoliation energies from first principles. *Nano Lett.* **18**, 2759-2765 (2018).

2 Chen, C. J. Introduction to Scanning Tunneling Microscopy: Second Edition. (Oxford University Press, 2007).

3 Britnell, L., Gorbachev, R., Geim, A. *et al.* Resonant tunnelling and negative differential conductance in graphene transistors. *Nat. Commun.* **4**, 1794 (2013).

4 Lyo, I. W. & Avouris, P. Negative differential resistance on the atomic scale: implications for atomic scale devices. *Science* **245**, 1369 (1989).

5 Xue, Y.-Q., Datta, S., Hong, S.-H., *et al*. Negative differential resistance in the scanning-tunneling spectroscopy of organic molecules. *Phys. Rev. B* **59**, R7852(R) (1999).

6 Kim, K. S., Kim, T.-H., Walter, A. L., *et al*. Visualizing atomic-scale negative differential resistance in bilayer graphene. *Phys. Rev. Lett.* **110**, 036804 (2013).

7 Li, S.-Y., Liu, H., Qiao, J.-B., Jiang, H., & He L. Magnetic-field-controlled negative differential conductance in scanning tunneling spectroscopy of graphene npn junction resonators. *Phys. Rev. B* **97**, 115442 (2018).

8 Yin, L.-J., Yang, L.-Z., Zhang, L., Wu, Q., Fu, X., Tong, L.-H., Yang, G., Tian, Y., Zhang, L., & Qin, Z. Imaging of nearly flat band induced atomic-scale negative differential conductivity in ABC-stacked trilayer graphene. *Phys. Rev. B* **102**, 241403 (2020).
